# Food-Safe Process for High Recovery of Flavonoids from Cocoa Beans: Antioxidant and HPLC-DAD-ESI-MS/MS Analysis

**DOI:** 10.3390/antiox9050364

**Published:** 2020-04-27

**Authors:** Said Toro-Uribe, Elena Ibañez, Eric A. Decker, Arley René Villamizar-Jaimes, Luis Javier López-Giraldo

**Affiliations:** 1 School of Chemical Engineering, Food Science and Technology Research Center (CICTA), Universidad Industrial de Santander, Carrera 27, Calle 9, Bucaramanga 68002, Colombia; saidtorouribe@gmail.com; 2 Foodomics Laboratory, Institute of Food Science Research (CIAL, CSIC-UAM), Nicolás Cabrera 9, 28049 Madrid, Spain; elena.ibanez@csic.es; 3 Department of Food Science, University of Massachusetts, Chenoweth Laboratory, 100 Holdsworth Way, Amherst, MA 01003, USA; edecker@foodsci.umass.edu; 4 School of Chemistry, Food Science & Technology Research Center (CICTA), Universidad Industrial de Santander, Carrera 27, Calle 9, Bucaramanga 68002, Colombia; arleyvil@uis.edu.co

**Keywords:** cocoa, polyphenols, solid–liquid kinetic extraction, antioxidants

## Abstract

Considering the increasing interest in the incorporation of natural antioxidants in enriched foods, this work aimed to establish a food-grade and suitable procedure for the recovery of polyphenols from cocoa beans avoiding the degreasing process. The results showed that ultrasound for 30 min with particle sample size < 0.18 mm changed the microstructure of the cell, thus increasing the diffusion pathway of polyphenols and avoiding the degreasing process. The effect of temperature, pH, and concentration of ethanol and solute on the extraction of polyphenols was evaluated. Through a 2^4^ full factorial design, a maximum recovery of 122.34 ± 2.35 mg GAE/g, 88.87 ± 0.78 mg ECE/g, and 62.57 ± 3.37 mg ECE/g cocoa beans, for total concentration of polyphenols (TP), flavonoids (TF), and flavan-3-ols (TF3), respectively, was obtained. Based on mathematical models, the kinetics of the solid–liquid extraction process indicates a maximum equilibrium time of 45 min. Analysis by HPLC-DAD-ESI-MS/MS showed that our process allowed a high amount of methylxanthines (10.43 mg/g), catechins (7.92 mg/g), and procyanidins (34.0 mg/g) with a degree of polymerization >7, as well as high antioxidant activity determined by Oxygen Radical Absorbance Capacity (1149.85 ± 25.10 µMTrolox eq/g) and radical scavenging activity (DPPH^•^, 120.60 ± 0.50 µM Trolox eq/g). Overall, the recovery method made possible increases of 59.7% and 12.8% in cocoa polyphenols content and extraction yield, respectively. This study showed an effective, suitable and cost-effective process for the extraction of bioactive compounds from cocoa beans without degreasing.

## 1. Introduction

Flavanols are the most abundant substances of polyphenols found in cocoa, with a degree of polymerization ranging from monomers to polymeric proanthocyanidins [1]. Therefore, unfermented cocoa bean is composed of 1.3–3.3% methylxanthines [2] and of about 6% condensed flavan-3-ols [3]; as a result, it is has been listed as the 4th richest dietary source of polyphenols [4]. Cocoa is one of the top crops in Colombia in terms of economic impact, with a national production record of 59,665 tons in 2019 according to National Cacao Producers Federation. There is also a government initiative aiming at a switch from cocaine to cocoa and to join forces with the private sector to enhance Colombia’s competitiveness at an international level, which has been established in the comprehensive national program for the replacement of illegal crops [5].

Polyphenols have gained increasing attention as supplements and additives in functional foods due to their nutraceutical properties and beneficial health properties. The recovery of polyphenols from cocoa and its by-products through several technologies such as maceration [6], microwave [7], and pressurized liquid extraction [8] has been previously reported. Most of them use powder cocoa beans obtained from processing operations consisting mainly of (a) drying and particle size reduction pretreatment, (b) degreasing, and (c) recovery of secondary metabolites. During the pretreatment stage, low drying temperatures and short times are preferred to avoid oxidation of flavonoids while small particle size is suggested to increase mass transfer.

The total fat content of whole cocoa beans is over 50% (on a dry basis) [9], which constitutes a barrier for the release of polyphenols from the cells; therefore, a degreasing process is commonly employed that can include pressing of the beans, or using a solvent extraction (i.e., hexane, chloroform, petroleum ether or other non-polar solvents) [10,11] to achieve a final fat content lower than < 12 wt.% [12]. When defatting using solvents, an additional step for residual solvent removal is thus needed. Supercritical fluid CO_2_ can be used, which leaves no residue in the final product, but it is more expensive [13].

During the last stage, the extraction yield can vary not only due to the pre-treatment step, but also due to the type of solvent, contact time, temperature, solid to solvent ratio, the structure of the solid matrix and pH [14]. Although several solvents can be used to extract cocoa polyphenols, polar solvents approved by food regulations agencies (i.e., FDA and EFSA) for human consumption (that is, water, ethanol or a mixture of both) are preferred.

Some patented processes can be found in the literature for producing cocoa polyphenol concentrate; these include a series of sequential steps consisting on inhibition of enzymatic browning (blanching with hot water or steam), pressing and supercritical CO_2_ extraction for fat removal or degreasing with hexane over 6 h [13], reduction of particle size (<500 um) using cryogenic milling (under −5 °C), and extraction of polyphenols using hot water [13], 60% 2-propanol [15], acetone/water/acetic acid [16] and aqueous methanol [17].

These are good examples showing that no single universal extraction processes can be employed for the extraction of polyphenols from different plant sources [18]. Therefore, the goal of the present work was to establish the optimal experimental conditions to enhance higher recovery of polyphenols from cocoa beans without degreasing. Thus, a suitable method that was reproducible for large-scale production (0.05–10 L), cheap, used food grade solvents and reliable for food applications was developed based on ultrasound-assisted solid-liquid extraction using aqueous ethanol. The best operational conditions for the pre-treatment process (drying and particle size reduction to avoid the degreasing) and for the recovery of polyphenols were established. In addition, chemical characterization and antioxidant activity of the cocoa polyphenol extract were also studied.

## 2. Material and Methods

### 2.1. Reagents

All the chemicals used were analytical or reagent grade and were not purified further. Folin-Ciocalteu reagent, gallic acid, sodium carbonate, ascorbic acid, l-cysteine, sodium phosphate monobasic monohydrate (NaH_2_PO_4_·H_2_O), disodium hydrogen phosphate (Na_2_HPO_4_), caffeine, and theobromine were obtained from Sigma Aldrich (St. Louis, MO, USA). (+)-Catechin hydrate (≥99%; ASB-000003310), (−)-epicatechin (≥99%; ASB-00005127), procyanidin B2 (≥90%; ASB-00016231) were purchased from ChromaDex Inc. (Irvine, CA, USA). Acetonitrile, methanol, (HPLC-grade), ethanol, *n*-hexane, citric acid and formic acid were acquired from Merck (Merck, Germany). Deionized water (18 MΩ/cm) from an Aqua Solution system (Aqua solution, Inc. Jasper, Georgia, USA) was used for the preparation of all solutions.

### 2.2. Pre-Treatment of Cocoa Beans

Fresh cocoa pods (Trinitary, clone ICS 39) were collected at Villa Santa Monica (San Vicente de Chucurí, Santander, Colombia) and immediately protected from light and transported on ice to CICTA Lab for processing. Beans were removed manually from the pods using a knife, cleaned and separated from the pulp (mucilage) using a mucilage remover (Penagos Ltd.a, Colombia). After that, beans were heat-treated (96 °C for 6.4 min) with an enzyme inhibition solution (70 mM·L-ascorbic acid and l-cysteine, ratio 1:1 *v*/*v*) until reaching a polyphenol oxidase inhibition up to 93%. The inactivated beans were frozen at −20 °C and used for further analysis.

#### 2.2.1. Drying and Milling Process

Drying and milling processes were evaluated as follows:

(a) Drying: Beans after polyphenol oxidase (PPO) inactivation were used immediately to evaluate the effect of drying technology on the total polyphenol content. To do so, beans were chopped (cross section of 50 × 30 mm^2^). Then, beans were a) oven dried (FD 23, Binder, Germany) at both 50 °C and 70 °C at atmospheric pressure, and b) freeze-dried (Labconco Corp., Kansas City, MO, USA) at −84 °C processing temperature and 13 Pa constant pressure in the drying chamber to obtain final humidity < 4%. The moisture content was evaluated by AOAC method 931.04 (AOAC, 1990).
(b) Milling: As a strategy to avoid the use of a non-GRAS (Generally Recognized as Safe) solvent for removing the fat from cocoa beans, different particle size distributions and ultrasound time were evaluated as a function of total phenol content. Dried beans were milled at max speed at −20 °C in N_2_ environment for 30 s during three cycles (Grindomix GM 200, Retsch, Haan, Germany). The milled samples were sieved through steel mesh (W.S. Tyler, Mentor, OH, USA) with a sieve shaker (Gilson, Screen Co., USA) and fractionated in three groups: sieved and retained on 20 to 40-mesh (sample 1); 40 to 80-mesh (sample 2); and 80 to 200-mesh (sample 3). After that, the powdered sample was immersed in 50% aqueous ethanol and ultrasonicated (35 kHz, ice bath at 4 °C, Elma, Ultrasonic LC20H, Germany) for several intervals of time. A defatted cocoa sample (<5 wt.%) was employed as a control sample. To do so, the cocoa bean powder (1.0 g) was three times defatted with *n*-hexane (10 mL, extraction in an ultrasonic bath at 25 °C for 15 min). The resulting powder was dried overnight at room temperature.

In all experiments, a standard polyphenol extraction step was carried out as follows: 1 g of sample was added to 60 mL of a mixture of 50% ethanol and water (*w*/*w*) at 50 °C with stirring at 300 rpm for 30 min using a magnetic stirrer hotplate (IKA C-MAG HS7, Germany) with temperature being monitored with a thermocouple (IKA ETS-D5, Germany). The resulting extract was centrifuged (5000× *g*, 4 °C, 20 min), then the supernatant was filtered through 0.45 µm hydrophilic PTFE filter (Millipore, Milford, USA), and the total polyphenol content was measured immediately as detailed in Section 2.4.1.

#### 2.2.2. Scanning Electron Microscopy

Scanning electron microscopy (SEM) was used to evaluate the microstructure of (a) raw cocoa beans, (b) cocoa beans with reduced PPO activity, and (c) cocoa beans with reduced PPO and treated by ultrasound. Bean samples cut into longitudinal and transversal sections were mounted on aluminum stubs with double-sided carbon adhesive tape and observed using the XL-30 Environmental SEM (Philips, USA) at 25 kV accelerating voltage with the BSE (backscattered electron) detector. The images were stored in TIFF format at 712 × 484 pixel in grayscale with brightness values between 0 and 255 for each pixel constituting the image.

### 2.3. Solid–Liquid Extraction of Polyphenols

Extraction temperature, pH, solute/solvent ratio, and ethanol/water ratio were evaluated as the major factors that can affect the extraction yield. pH was adjusted with 1 M citric acid. The combination of these factors was modeled through a surface design consisting of 2^4^ + four replicates at the central point + triplicates at the start point. Low and high levels for the different factors were, as follows: Temperature (40, 60 °C); pH (3, 5); solute/solvent ratio (*w*/*v*) (1/60, 1/30); ethanol/water ratio (*v*/*v*) (25/75, 75/25).

The resulting extracts were centrifuged at 5000× *g*, 4 °C for 15 min and the supernatant was collected and filtered using Whatman Nº1 filter paper (Whatman, Inc., USA), and used for further experiments. At maximum extraction conditions, large-scale recovery (up to 10 L) controlled by Bioflow-110 bioreactor (New Brunswick Scientific, Enfield, CT, USA) was used to prove that the scale up of the process is possible.

### 2.4. Determination of Total Polyphenol, Total Flavonoids, and Total Flavan-3-ols Content

#### 2.4.1. Total Polyphenol Content by Folin-Ciocalteu

The total polyphenol (TP) content was assayed using the Folin-Ciocalteu reagent according to Singleton et al. [19] with modifications as follows. The reaction was initiated by the addition of 50 µL of the sample with 1.5 mL of 10-fold diluted Folin-Ciocalteu reagent. After 5 min, 1.5 mL of 7.5% (*w*/*v*) sodium carbonate was added and vortexed for 10 s. The reaction medium was stored in the dark for 1 h at 25 °C. Absorbance was measured at 765 nm (Genesys 20; Thermo Scientific-Fisher, Waltham, MA, USA) against a blank sample. A calibration curve of gallic acid (ranging from 0.01–0.8 mg/mL, *r*^2^ = 0.99) was prepared, and the results were expressed as mg gallic acid equivalents (GAE) per gram of dried cocoa beans.

#### 2.4.2. Total Flavonoid Assay

Total flavonoid (TF) content was measured according to Zhishen et al. [20]. Sample (500 µL) was added to 5 mL volumetric flask containing 2 mL H_2_O followed by addition of 0.15 mL 5% NaNO_2_. After 5 min, 0.15 mL 10% AlCl_3_ was added and 1 min after, 1 mL 1M NaOH was added. The total volume was made up to 5 mL with H_2_O. The reactants were mixed well and stored in the dark for 15 min at 25 °C. Absorbance was measured at 510 nm (Genesys 20; Thermo Scientific-Fisher, Waltham, MA, USA) against a blank sample. Total flavonoid content was expressed as mg (−)-epicatechin equivalents (ECE) per gram of dried cocoa beans.

#### 2.4.3. Total Flavan-3-Ol Assay

Total flavan-3-ol (TF3) content was determined by the vanillin-H_2_SO_4_ assay as described by Sun [21]. The reaction consisted of 1 mL sample in methanol with 2.5 mL of 1% vanillin in methanol and 2.5 mL of 9N H_2_SO_4_. The reaction medium was well mixed at 30 °C and allowed to react for 15 min. Absorbance was measured at 500 nm (Genesys 20; Thermo Scientific-Fisher, Waltham, MA, USA) against a blank sample. Total flavan-3-ol content was expressed as mg (−)-epicatechin equivalents (ECE) per gram of dried cocoa beans.

### 2.5. Kinetic of Solid–Liquid (S-L) Extraction of Polyphenols

Once the optimum S-L extraction conditions were selected, the extraction kinetics were evaluated by plotting the concentration of the isolated target analyte versus time. Aliquots (100 µL) of each sample were taken out at various times to measure the total concentration of polyphenol, flavonoid, and flavan-3-ol. The extraction curves were adjusted by several kinetic models previously documented [22,23,24,25,26].

### 2.6. Characterization by HPLC-DAD-ESI-MS/MS

HPLC analysis was performed on a Shimadzu (LC-2030 LT Series-i, USA) equipped with a photodiode detector, solvent degasser, quaternary pump, autosampler with temperature control, and thermostat column compartment. The separation was achieved using a C18-phenyl column (4.6 × 50 mm, 2.5 µm particle size) (Xbridge^®^, Waters, USA) protected with a security guard obtained from Phenomenex (AJ0-8788, Phenomenex, Torrance, CA, USA). The procedure consisted of acidified water (water/formic acid, 99.9:0.01 *v*/*v*) (solvent A) and acidified acetonitrile (acetonitrile/formic acid, 99.9:0.01 *v*/*v*) (solvent B). The optimized linear gradient was as follows: 0–8 min, 2% B; 8–37 min, 10% B; 37–40 min, 0% B and 2% B for 10 min. The flow rate was 0.8 mL/min, and the temperature was 60 °C. The detector acquisition was 190–800 nm. The calibration curves for caffeine, theobromine, catechins, and dimer B2 were made from commercially available analytical standards (*r*^2^ = 0.99). Oligomeric procyanidin calibration curve was performed from isolated fractions (*r*^2^ = 0.98) according to Toro-Uribe et al. [27]. All the results are expressed as mg of sample per g of cocoa beans (dry matter basis).

The mass detector was an Agilent 6320 Ion Trap LC/MS (Agilent Technology, Waldbronn, Germany) equipped with an ESI source and ion trap mass analyzer which was controlled by the 6300 series trap control software (Bruker Daltonik GmbH). MS/MS analyses were carried out to obtain the structural information of the separated compounds. Mass spectrometer was operated under positive and negative ESI mode, nebulizer pressure, 40 psi; dry gas, 12 L min^−1^; dry temperature, 350 °C, and mass spectra recorded from 90 to 2200 *m/z*. MS characterization features were analyzed using extraction ion compound tool and commercial standards. It was also consulted METLIN and HMBD databases for matching exact mass.

### 2.7. Antioxidant Assays

Oxygen radical absorbance capacity (ORAC) assay described by Huang et al. [28] was carried out as follow: 96 well microplates were filled with 50 µL of the daily working fluorescein solution (4 × 10^−3^ mM in 75 mM phosphate buffer, pH 7.4), 50 µL of samples at a known concentration, and incubated at 37 °C for 10 min in a microplate reader (Synergy HT Multi-Detection, Biotek Instruments, Inc. Winooski, VT, USA). The reaction was initiated by the addition of 50 µL of 2,2′-Azobis(2-methylpropionamidine) dihydrochloride (221 mM in 75 mM phosphate buffer, pH 7.4) and the fluorescence decay was monitored kinetically for 2 h, using emission and excitation wavelength of 485 nm and 528 nm, respectively. A calibration curve was prepared with 12.5–375 µM Trolox (*r*^2^ = 0.99). Results are expressed as µM Trolox equivalents per g of cocoa beans (dry matter basis) as follows:

$$ORAC=\frac{AUC_{sample}-AUC_{blank}}{AUC_{Trolox}-AUC_{blank}}*k*\frac{molarity\;of\;trolox}{weight\;of\;sample}$$

where *k* is the sample dilution factor, and AUC is the area below the fluorescence decay curve of the sample, blank, and Trolox, respectively. The area under the curve of normalized data was calculated using GraphPad Prism v. 6.0 (GraphPad Soft. Inc., La Jolla, San Diego, CA, USA).

The reducing ability of antioxidants toward the DPPH (2,2-diphenyl-1-picrylhydrazyl) radical was measured according to Brand-Williams et al. [29]. DPPH methanolic solution (684.7 µM) was adjusted with methanol until an absorbance of 0.550 ± 0.01 units at 517 nm (Genesys 20, Thermo Scientific, Waltham, MA, USA) was obtained. Samples (100 µL) were incubated with 1.45 mL of this DPPH solution for 60 min in the dark. A calibration curve was prepared from 1.95–150 µM Trolox (*r*^2^ = 0.99). Results are expressed as µM Trolox equivalent per g of cocoa beans (dry matter basis).

### 2.8. Statistical Analysis

All the experimental measurements were repeated at least three times, and data were expressed as the mean ± standard deviation. Statistical analysis was done using Statistica 7.1 (Stat-Soft Inc., USA). The analysis of variance (ANOVA) and *F*-test were used to evaluate the influence of the factors and their interactions on the experimental designs. ANOVA one-way and Tukey’s multiple range test was also conducted at a 5% level of significance. The kinetic constants in this study were determined from experimental data using non-linear regression employing Quasi-Newton method and least squares as custom loss function (0.0001 for both start value and initial step size). The response surface methodology consisting of full factorial central composite rotatable design with four replicates at the central point was conducted according to a completely randomized model. A second-order polynomial equation was used to fit the experimental data as follows:

$$Y=\beta_{0}+\overset{k}{\underset{i=1}{\sum }}\beta_{i}X_{i}+\overset{k}{\underset{i=1}{\sum }}\beta_{ii}X_{i}^{2}+\overset{k-1}{\underset{\begin{array}{c} i=1\\ i<j \end{array}}{\sum }}\overset{k}{\underset{j=2}{\sum }}\beta_{ij}X_{i}X_{j}$$

where *Y* is the predicted factor, *β*_0_ is the value of the fitted response to the design, *β_i_*, *β_ii_*, and *β_ij_* are the coefficients of linear, quadratic, and cross product terms, respectively.

The performance of full factorial central composite design method was measured by *r* and *r*^2^. Experimental runs were also randomized to evaluate the concordance of experimental data and predicted values; therefore, the root mean squared error (RMSE) was calculated as follows:

$$RMSE=\sqrt[{2}]{\frac{\sum_{i=1}^{n}{\left(y_{i}-\hat{y_{i}}\right)}^{2}}{n}\;}$$

where $y_{i}$ and $\hat{y_{i}}$ is the measured value and predicted value by the model, respectively. In addition, *n* is the number of the set data.

## 3. Results and Discussion

### 3.1. Effect of Drying Temperature, Particle Size and Non-Degreasing Process on the Concentration of Total Phenols

#### 3.1.1. Drying Technology

Beans with reduced enzyme activity were manually chopped, and the total phenol content was evaluated after (a) oven-air drying at 50 °C and 70 °C, and (b) freeze-drying. The initial moisture content in a fresh unfermented bean was 40.1 wt.% and after drying process the final moisture content was between 1.4–3.2 wt.%. Table 1 summarizes the effect of drying process on TP content. Therefore, high temperature air drying led to a great extent of dehydration levels. Visual evidence showed that dried beans were slightly grey/purple color, which is characteristic of dried unfermented cocoa beans with high procyanidin content [12]. However, drying at 50 °C for 20 h resulted in TP content 10% lower than drying at 70 °C for 3 h (*p* < 0.05), which showed that polyphenols were more sensitive to drying time than temperature. No significant differences in TP amount was observed between freeze drying and oven-dried at 70 °C treatments, which was also reported by Kothe et al. [30], who found that when employing high temperatures and short times (100–160 °C for 30 min), different reactions can occur, such as configuration rearrangement of flavan-3-ols and epimerization, which could led to the formation of new flavan-3-ol products with higher phenol activity. Thereby, oven-air drying at 70 °C was chosen as drying technology for further experiments. Moreover, air drying is a more cost-effective solution (and faster) than freeze-drying.

#### 3.1.2. Impact of Particle Size on Extraction Yield

After the PPO enzymatic inhibition and drying steps, the samples were milled at low temperature for a short time, thus avoiding the fat melting during the reduction of particle size. In an attempt to discriminate which sizes led to better performance on extraction yield, three distinct samples varying on particles size were evaluated as follow: (S_1_) > 0.42 mm, (S_2_) 0.42–0.18 mm, and (S_3_) < 0.18 mm. The results showed that the best treatment was S_3_ with a TP recovery of 50 mg GAE/g being 2× and 1.2× fold higher than S_1_ and S_2_, respectively. This study reinforces the idea that there is an inverse relationship between the particle size and the extraction yield. This behavior was also observed by Sun et al. [31], who found that extraction yields were 6 fold higher when particle size decreased from 2 to 0.074 mm.

#### 3.1.3. Conditions to Avoid the Degreasing Process

**Figure 1:**
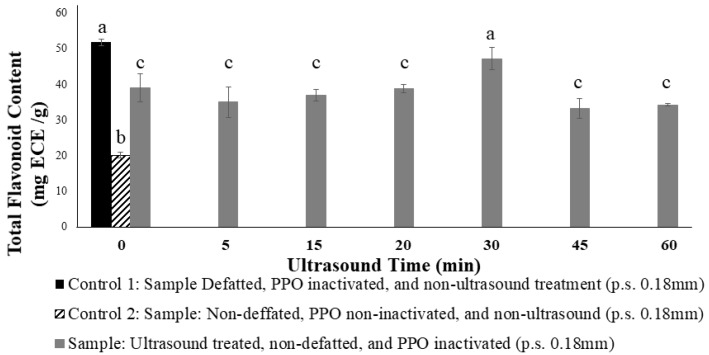
Impact of time of ultrasonic treatment on total polyphenolic amount. Means with different letters were significantly different by Tukey (*p* < 0.05).

Non-defatted samples and defatted samples with a total fat content of 58.5% and 2.5%, respectively, were used for these experiments. In prior assays, results showed that TP extraction from the defatted samples was 60% higher than in non-defatted samples, which confirms that fat is a barrier for the diffusion of the bioactive compounds from the solid phase to the extractor solvent. Since one of the aims of the present work was to avoid the use of toxic organic solvents, ultrasonic treatment was selected as an alternative clean extraction technology. Thus, non-defatted cocoa samples were placed in a test tube and mixed with 50% ethanol (1/60 *w*/*v* ratio solute/solvent) and ultrasonicated (35 kHz, 4 °C). The effect of ultrasonic time (0–60 min) was tested as a function of TP amount. As can be seen in Figure 1, ultrasound increases the polyphenols extraction efficiency up to a 30 min. This is because the cavitation forces improving the polarity of the system; moreover, the bubbles in the liquid/solid extraction can explosively collapse and generate localized pressure causing plant tissue rupture [32], thus increasing the mass transfer rate. Figure 1 also shows that samples ultrasonicated for 30 min had no significant difference with the defatted control (*p* < 0.05). Thereby, the ultrasonic bath for 30 min was chosen as optimal value for further experiments.

#### 3.1.4. Microscopy Analysis

**Figure 2:**
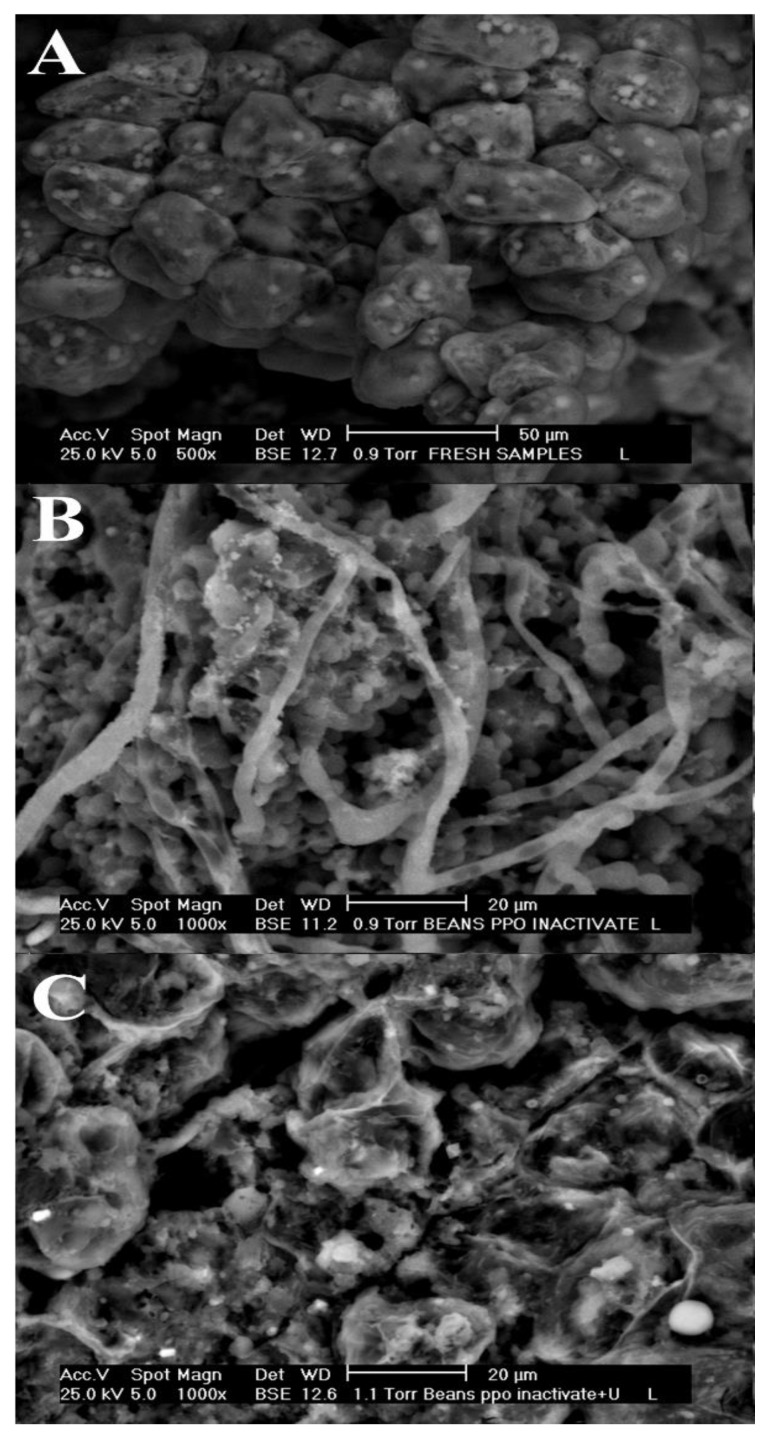
Microscopy images for the microstructure of (**A**) non-treated cocoa bean, (**B**) beans after PPO inhibition, and (**C**) beans after PPO inhibition and ultrasound treatment.

Figure 2 shows SEM photographs confirming the mechanical effect of ultrasound on cell wall structure. As can be observed, non-treated samples had intact cell walls which were oval-shaped and not fractured, as well as appearing more solid and denser due to the cellular contents remaining embedded in the cell (Figure 2A). In general, the cocoa bean is formed of parenchyma cells (containing cocoa butter and proteins), which represent over 80% of the mass [33], and polyphenols and alkaloids, which are placed into the vacuoles [34]. Physical changes were observed in the structure of cocoa starch granules that become larger, smooth and fibrous, quite likely as produced by heat treatment (Figure 2B). This behavior could be the result of enzyme heat treatment, which opens up the cell and favors diffusion of bioactive compounds from the matrix into the extraction medium, as well as to the mobilization of proteins and polyphenols and redisposition of fat within the cell [34].

The morphological differences are more evident in Figure 2C. Smaller fragments were dispersed within the cell, and the microstructure was more porous, which could be the result of cell disruption and changes in the intercellular spaces. Hence, changes in the cell might increase the permeability and thus diffusion of polyphenols out of the cells. In accordance with our results, Rostagno et al. [35] found that disruption of tissue and cell walls are most efficient when heat treatment together with ultrasound are used, which resulted in a greater penetration of solvent into the sample matrix, increasing the contact surface between solid and liquid phase, and as a result, the solute quickly diffuses from the solid phase to the solvent. The observation of this study confirms that the enzymatic inhibition by heat treatment, milling process, and ultrasounds play a significant role in the change of the internal structure of cocoa beans, thus enhancing a high concentration of polyphenols while avoiding the defatting process.

### 3.2. Solid–Liquid Extraction of Polyphenols from Cocoa Beans

As mentioned, temperature, solute/solvent ratio, ethanol/water ratio, and pH were evaluated through a surface experimental design. As can be seen in Table 2, the concentration of ethanol had a strong impact on response factors, for instance, TP, TF and TF3 varied from 41.3–107.6 mg GAE/g, 15.2–86.0 mg ECE/g, and 21.7–59.8 mg ECE/g, respectively, throughout the ethanol concentration range (0–100%). Moreover, TP yield increased with increasing extraction temperature, i.e., extraction at 60 °C (25% ethanol, pH 5, 1/60 solvent/solute ratio) was 1.12 fold higher at 40 °C. Maximum values for TP (107.6 mg GAE/g), TF (86.0 mg ECE/g), and TF3 (59.8 mg ECE/g) were observed at different levels of factors, which indicate that combination of factors plays a key role in the extraction process.

**Figure 3:**
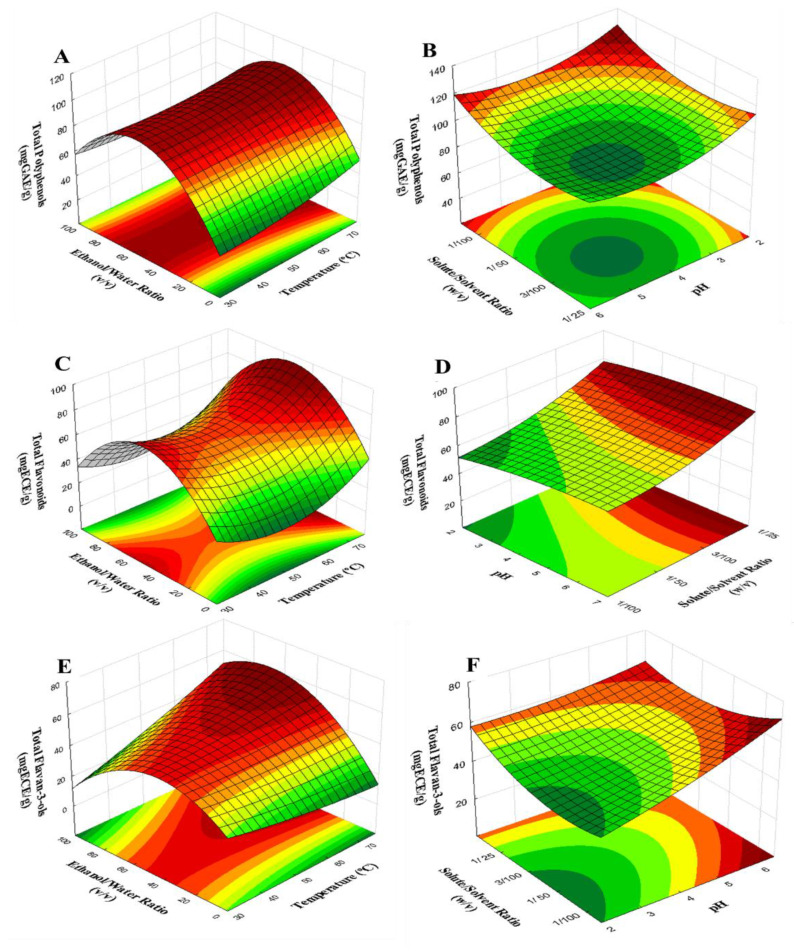
Surface response as function for Temperature vs. Ethanol concentration, and pH vs. solute concentration for the recovery of total polyphenols (**A**,**B**), total flavonoids (**C**,**D**) and total flavan-3-ols (**E**,**F**).

Results were fitted to a quadratic model (Equations (S1)–(S3)) and the 3D surface graphs for dependent variables were plotted (Figure 3). Thus, analysis of variance summarized the observations and differences among the studied factors (Table S1). ANOVA showed that the recovery of bioactive compounds was mainly dependent on the linear effect of temperature (T) and pH, linear and quadratic effect of ethanol/water (EW) ratio, and solute/solvent (SS) ratio. Besides, the interactions of T × EW, and EW × pH for total polyphenol content; SS × T, and SS × EW for total flavonoid concentration; and T × SS, T × EW, SS × pH, and EW × pH for total flavan-3-ol amount were also significant (*p* < 0.05). ANOVA analysis also showed that the selected quadratic model adequately represented the extraction process. Therefore, the model has a good coefficient of multiple determination of $r^{2}=0.957$, $r_{adj}=0.941$; $r^{2}=0.954$, $r_{adj}=0.937$; and $r^{2}=0.905$, $r_{adj}=0.872$ for total polyphenol, flavonoid and flavan-3-ols content, respectively. Overall, higher accordance regression fit values mean that the model explained most of the variability in the responses.

Thereby, the maximum experimental conditions were enhanced with 50/50 *v*/*v* ethanol/water ratio, 1/120 *w*/*v* solute/solvent ratio, pH 6 at 70 °C for a predicted recovery of 117.87 ± 16.68 mgGAE/g, 85.22 ± 18.51 mgEC/g, and 76.86 ± 15.98 mgECE/g, which were in agreement with the experimental data with 122.34 ± 2.35 mg GAE/g, 88.87 ± 0.78 mgECE/g, and 62.57 ± 3.37 mgECE/g, for TP, TF, and TF3, respectively.

#### Effect of Independent Factors on the Recovery of Total Polyphenols and Total Flavonoids

As can be seen in Figure 3, the significant factors evaluated had a parabolic behavior on the extraction of TP, TF, and TF3. For instance, it was confirmed that temperature promoted faster diffusion rate, better mass transfer, reduce viscosity and surface tension (Figure 3A,C,E), which is in agreement with Cacace and Mazza [36]. Indeed, this factor had a linear effect until it reached a maximum, after which the extraction yield decreased. This phenomenon is explained by the softening of plant tissue at high temperature, while at the same time, it weakens phenol-protein and phenol-polysaccharide interactions [37]. Moreover, results showed that higher diffusion rate is enhanced at lower ratio solute/solvent, which is confirmed by the significance of linear and quadratic effect for both total phenol and total flavonoid content. Also, Fick´s second law of diffusion predicts this phenomenon, that is, a final equilibrium between the concentration of solute in the solid matrix and in the bulk solution after a certain time [37].

The influence of ethanol concentration (Figure 3A,C,E) suggested that the extract contains polyphenols with different polarities, which also confirms the principle “like dissolves like” The lowest concentration of water or ethanol were not efficient for the extraction of flavonoids from cocoa beans. The maximum yield was enhanced at 50% ethanolic aqueous solution. Previous works are consistent with our results, reporting a maximum extraction yield of polyphenols with an ethanol/water ratio of 40–50% [18,38], which is because the concentration of ethanol influences the velocity-transfer kinetic and the thermodynamics of the process [38].

In addition, the interaction of pH with the solvent (Figure 3B,D,F) plays an essential role in controlling adsorption/desorption rate (i.e., changing the surface charge of adsorbent, the degree of ionization and speciation of adsorbate during adsorption) [39], and an impact on the pKa value for the polyphenols. In fact, lower solvent acidity allowed the highest recovery of flavonoids, because acid could hydrolyze the cell wall and thus facilitates the diffusion of the phenolic compounds. However, at higher concentrations of ethanol, a reduction on the extraction yield was observed. Similar results show that the amount of acid in the extraction solvent increases concentration by increasing the stability of the phenolics during extraction and increasing their dissolution [37].

In addition, significant differences (*p* < 0.05) among control samples and sample at optimum conditions were found; thereby, TP, TF, and TF3 were 59.7, 59.8, and 57.8% higher, respectively, when employing the optimized S-L extraction method (Table 2).

### 3.3. Extraction Kinetics Parameters

Given the maximum conditions for the recovery of polyphenols, different extractions were carried out to evaluate the equilibrium time and kinetic parameters as a function of TP, TF, and TF3 content.

**Figure 4:**
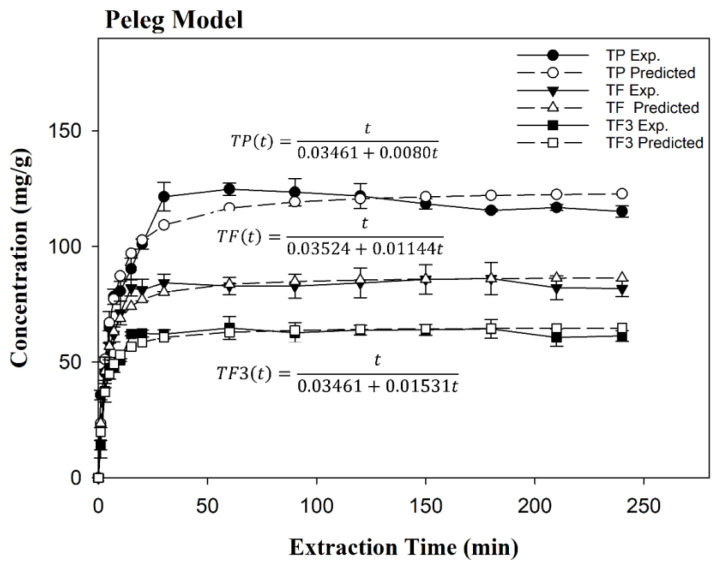
Experimental and calculated extraction curves for a total polyphenol (TP), total flavonoids (TF), and flavan-3-ols content. E (TF3). Extraction rate constant based on Peleg model and solid–liquid extraction at optimal conditions.

The study of equilibrium time plays an essential role in economizing the energy and cost of the industrial process [37], thus improving the accuracy of the procedure and the quality of the final product. Table 3 summarizes the kinetic parameters, RMSE and the accordance of the model (*r*) for all the mathematical models selected, which were previously used to model the solid-to-liquid extraction of bioactive compounds. Overall, TP, TF, and TF3 content had the lowest *r* and highest RMSE for those equations that consider the extraction is occurring in one continuous step, i.e., Equations (1), (2), (4), and (5). Goodness of fit of the model and lower standard deviation of residuals were obtained for the two models that represent the recovery of polyphenols on two different rates (sorption/desorption), for instance, Equation (3) and Equation (6). These equations represent the kinetic process of a liquid/solid system based on the solid capacity. As can be seen in Figure 4, the extraction curve shape had a faster extraction rate followed by a slower extraction rate and asymptotically approached the equilibrium concentration. Generally, the temperature enhances the solubility of polyphenols in the solvent [40], but the rate of extraction yield decreased progressively due to the exposure to both higher temperature and time extraction, which confirms that polyphenols are thermosensitive compounds [41].

Results suggested that the Peleg model (Equation (3)) proved to be most suitable to model the solid–liquid extraction kinetics for the dependent variables (*r* ≥ 0.98 and RMSE ≤ 0.71). In fact, *k*_1_ and *k*_2_ represent the extraction rate constant and constant of extraction extent, respectively [42]. Table 3 shows that *k*_1_ was similar in all the cases, but *k*_2_ increased by TF3 ~ TF >> TP, which is related to its maximum equilibrium concentration ($C_{t\to \infty }$). Thereby, the equilibrium extraction time were 45 min, 39 min and 34 min for TP, TF, and TF3 content, respectively. Comparison of these data with those of previous authors shows that our optimized extraction method was 2.7×, 4.4×, 6.0×, and 2.7× faster than the polyphenol recovery of cocoa beans [43], grape seed [42], and mango kernel [44], respectively.

In addition, a large-scale extraction process was also assayed. The results showed that recovery from 0.05 to 5 L did not significantly impact extraction yield (*p* < 0.05), but the extraction at 10 L was 25.7 ± 0.9% lower for TP content while was 23.0 ± 6.9% and 5.3 ± 2.6% higher for TF and TF3 amount, respectively. These differences could be due to diffusional changes as a result of the scaling up the process.

In general, this work confirmed the importance of studying the impact of extraction parameters on both secondary plant metabolites, thereby increasing the yield of extraction. In terms of going beyond the highlights, the optimized ultrasound-assisted solid–liquid extraction not only allowed a high concentration of both total polyphenols and flavonoids but also employed food-grade solvents, reduced the number of stages (i.e., avoiding the degreasing), the extraction time (<45 min), and therefore energy consumption. Thereby, the extraction process could be suitable for large-scale applications. For example, cocoa polyphenol extract can be used to enrich products in very high demand in the food and cosmetic industries.

### 3.4. Chromatographic and Antioxidant Analysis

**Figure 5:**
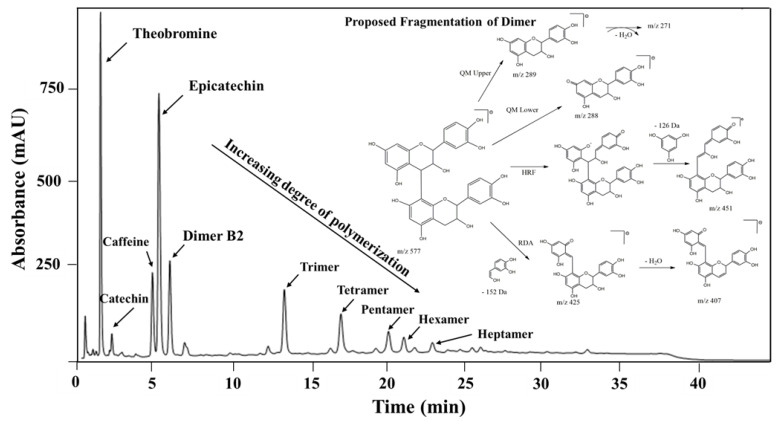
Chromatogram of cocoa extract and hypothetical electrospray ionization (negative mode) fragmentation pathway for procyanidin dimer. RDA, retro-Diels-Alder fission; QM, quinone methide cleavage; HRF, heterocyclic ring fission.

Identification of alkaloids, catechins and procyanidins in cocoa, were achieved by HPLC-DAD-ESI-MS/MS. The chromatographic method allowed not only the detection of main alkaloids but also the procyanidins with different degree of polymerization up to 7 (Figure 5). As can be seen in Table 4, the cocoa extract obtained at maximum conditions contained 65% (*w*/*w*) procyanidins followed by methylxanthines (20% *w*/*w*) and catechins (15% *w*/*w*), which is in agreement with previous authors [45,46]. Theobromine/caffeine ratio was 2.93, which can be used to classify hybrid genotypes [47]. Therefore, it was confirmed that the cocoa sample was of the Trinitarian variety. (−)-Epicatechin (7.30 mg/g) was the main catechin, being 11.8 fold higher than (+)-catechin, which was also confirmed by Romero et al. [48]. The major procyanidin in cocoa polyphenol extract was the Trimer C1 (11.9 mg/g) being 2.9×, 1.3×, 1.7×, 6.6×-fold higher than the dimer, tetramer, pentamer, and hexamer, respectively.

Our results are in agreement with previous works that reported that the main alkaloid and flavanol are the theobromine and (−)-epicatechin, but their concentrations vary considerably depending on the methodology of extraction and cocoa bean variety [49]. Theobromine and caffeine concentrations were in agreement with previous reports [8,15], but epicatechin and catechin amounts were 1.5× and 1.9×, and 2.0× and 2.3× fold higher than those reported by Kothe et al. [30] and Carrillo et al. [15], respectively. In general, procyanidin content was much higher than previous authors, i.e., dimer B2 was 2.0× and 1.5× -fold greater than procyanidin B2 previously reported by Kothe et al. [30] and Tomas-Barberán et al. [43], respectively.

In addition, the identification of oligomeric procyanidins was carried out by mass spectrometry in comparison with commercial standards and published literature. For instance, dimer B2 (B-type, EC-4β→8-EC-4β), trimer C1 (B-type, EC-4β→8-EC-4β→8-EC), tetramer, pentamer, hexamer and heptamer with a molecular ion [M − H]^−^ *m/z* 577, 865, 1153, 1441, 1729, 2019, respectively were identified. Characterization of larger polymers was not possible due to their low concentration, low ionization, peak signal dispersion, the formation of multiple ions, and limitations of the ion trap MS analyzer. Fragment patterns also suggested that various fragmentation mechanisms are involved in ESI such as quinone methide (QM), retro-Diels-Alder (RDA), as well as heterocyclic ring fission (HRF), which could take place on the extension unit or the terminal unit of the molecule [50]. For instance, the loss of a fragment with *m/z* 152, 170 and additional loss of water corresponding to RDA fission and loss of 288 Da corresponds to QM cleavage (Figure 5).

Among the antioxidant assays, our results showed that the cocoa extract had an ORAC of 1149.85 ± 25.1 µM Trolox eq/g. These values are higher than those reported by Hurst et al. [51] and Carrillo et al. [15], with TP in the range 58.0–61.7 and 45.3–70.0 mg GAE/g, and ORAC in the range 797.0–947.0 and 387.3–618.1 µM Trolox eq/g, respectively. Moreover, DPPH radical scavenging activity was 120.6 ± 0.5 µM Trolox eq/g (equal to 0.72 µM Trolox eq/mg cocoa extract), which was 2.4 fold higher than previously reported by Summa et al. [52]. These differences could be associated with the improved methodology of extraction at optimal conditions, thus enhancing both high composition and concentration of catechins (7.92 mg) and procyanidins (34.0 mg) with a DP ≥ 7.

## 4. Conclusions

An effective ultrasound-assisted solid–liquid process for extracting polyphenols from cocoa beans was optimized using an experimental design. The 2^4^ factorial design showed that all parameters studied were significant factors in affecting the polyphenolic content as well as enabling us to determine the optimal values for the extraction process. This optimization showed that the best conditions to obtain high polyphenol yield were 50% of ethanol, solid/solvent ratio of 1:120 *w*/*v*, pH 6 at 70 °C for a maximum equilibrium time of 45 min. Operating conditions to avoid degreasing and freeze-drying steps were also established, thus leading to a more cost-effective strategy. Overall, the process extraction allowed to increase on 59.7% and 12.8% of cocoa polyphenols amount and extraction yield, respectively. The extract rich in polyphenols could replace the synthetic antioxidants and could be used in the food and cosmetic industries. Our results suggest that ultrasound-assisted solid–liquid extraction is a suitable method for the recovery of cocoa polyphenols and could be used for the scaleup procedure for further research.

## Figures and Tables

**Table 1:** Effect of drying process on total polyphenol amount in cocoa beans.

Drying Method	Enthalpy of Sublimation (kJ/mol) *	Time (h)	Total Polyphenol Content (mgGAE/g)
Freeze Drying	51.00	48	43.99 ± 0.25 ^a^
Oven-air (50 °C)	41.69	20	38.96 ± 1.47 ^b^
Oven-air (70 °C)	40.84	3	43.20 ± 0.73 ^a^

Mean value ± standard deviation (*n* = 5) with similar letters mean are not significantly different by ANOVA One-way Tukey (*p* > 0.05). * The thermophysical property data for the determination of enthalpy of sublimation of pure compounds (water) was calculated thorough the libraries of the NIST ThermoData Engine (TDE) version 10.

**Table 2:** 2^4^ full factorial central composite rotatable design and experimental results for total polyphenol, total flavonoids, and total flavan-3-ols recovery from cocoa beans.

T (°C)	SS (*w*/*v*)	EW (*v*/*v*)	pH	TP (mgGAE/g)	TF (mgECE/g)	TF3 (mgECE/g)
60	1/30	25	5	78.22 ± 1.17	62.30 ± 1.75	35.07 ± 0.14
50	1/24	50	4	94.15 ± 0.50	79.64 ± 1.88	48.62 ± 0.59
50	1/40	50	2	98.86 ± 0.52	55.87 ± 2.60	39.46 ± 0.25
60	1/60	75	3	103.57 ± 1.25	57.48 ± 0.39	43.78 ± 0.90
40	1/30	75	3	92.23 ± 1.79	77.30 ±4.10	42.89 ± 0.27
60	1/60	25	3	91.59 ± 1.30	52.36 ± 0.39	40.74 ± 0.98
50	1/40	50	2	98.17 ± 1.10	53.84 ± 2.34	38.01 ± 0.13
40	1/30	75	3	95.81 ± 0.07	68.77 ± 3.51	40.75 ± 0.24
50	1/40	50	6	98.85 ± 0.65	66.45 ± 2.08	59.28 ± 0.98
50	1/40	50	4	95.96 ± 0.82	67.97 ± 4.16	51.26 ± 0.09
50	1/120	50	4	107.59 ± 0.52	53.74 ±0.77	55.21 ± 1.39
50	1/40	50	6	97.53 ± 0.26	64.31 ± 0.26	59.78 ± 1.54
60	1/30	75	3	97.90 ± 0.93	73.11 ± 2.35	47.46 ± 0.04
50	1/40	50	4	95.52 ± 0.61	63.01 ± 3.38	47.38 ± 0.93
40	1/30	75	5	81.23 ± 0.23	76.18 ± 4.30	50.28 ± 1.40
40	1/30	25	5	76.97 ± 1.29	61.21 ± 0.96	42.77 ± 0.28
40	1/60	75	5	91.56 ± 0.19	49.34 ± 2.72	42.19 ± 0.63
60	1/30	25	3	82.64 ± 0.13	59.53 ± 0.59	39.84 ± 0.07
60	1/60	75	5	89.26 ± 0.45	57.78 ± 0.39	58.68 ± 1.12
50	1/40	100	4	46.79 ± 0.44	15.18 ± 1.30	22.72 ± 0.37
40	1/60	25	3	84.58 ± 0.19	50.93 ± 3.12	38.83 ± 0.99
40	1/30	25	3	76.06 ± 0.69	55.74 ± 2.91	43.87 ± 0.21
40	1/60	25	3	84.45 ± 0.99	51.83 ± 3.52	40.37 ± 1.28
30	1/40	50	4	88.59 ± 0.13	74.73 ± 1.56	42.52 ± 0.14
70	1/40	50	4	100.04 ± 0.69	79.73 ± 4.14	58.04 ± 0.51
60	1/60	25	3	82.98 ± 2.23	55.26 ± 1.16	39.85 ± 0.85
40	1/30	25	5	78.95 ± 1.03	59.04 ± 3.69	46.46 ± 0.21
40	1/60	75	3	102.28 ± 2.25	46.02 ± 3.91	29.49 ± 0.61
60	1/30	25	5	81.21 ± 0.45	64.10 ± 3.88	38.59 ± 0.36
50	1/120	50	4	104.96 ± 0.91	55.91 ± 1.56	55.57 ± 0.56
60	1/30	75	5	83.48 ± 0.58	74.43 ± 4.27	55.98 ± 0.52
50	1/40	0	4	43.45 ± 1.22	22.40 ± 1.56	21.73 ± 0.09
40	1/60	25	5	76.43 ± 1.17	56.35 ± 2.34	41.86 ± 0.42
50	1/40	0	4	41.32 ± 0.22	17.09 ± 0.26	25.81 ± 0.65
30	1/40	50	4	89.97 ± 0.91	77.91 ± 2.60	41.31 ± 0.23
40	1/30	25	3	79.12 ± 0.67	58.51 ± 0.39	39.37 ± 0.25
60	1/30	75	5	84.29 ± 0.39	75.47 ± 4.26	52.63 ± 1.39
60	1/30	25	3	82.19 ± 0.39	59.47 ± 3.50	36.21 ± 0.39
60	1/60	75	5	89.01 ± 1.36	57.96 ± 1.16	54.35 ± 1.74
50	1/40	50	4	90.36 ± 0.48	69.19 ± 4.17	44.92 ± 0.84
60	1/60	25	5	85.11 ± 0.33	59.55 ± 1.95	44.08 ± 0.35
50	1/24	50	4	100.27 ± 0.05	86.05 ± 4.53	53.69 ± 0.25
70	1/40	50	4	97.97 ± 0.26	82.05 ± 2.08	58.80 ± 0.84
60	1/60	75	3	95.49 ± 2.51	53.52 ± 0.78	46.89 ± 1.50
60	1/30	75	3	98.69 ± 0.57	72.95 ± 3.50	49.99 ± 1.22
50	1/40	50	4	93.54 ± 1.13	64.19 ± 1.30	45.78 ± 0.77
40	1/30	75	5	81.22 ± 0.49	70.55 ± 2.15	47.68 ± 0.77
50	1/40	50	4	90.52 ± 0.30	65.81 ± 4.16	50.50 ± 0.79
50	1/40	50	4	91.27 ± 0.17	64.09 ± 3.90	50.16 ± 0.19
40	1/60	25	5	77.75 ± 0.91	51.53 ± 0.39	41.35 ± 1.01
40	1/60	75	3	99.96 ± 1.39	45.00 ± 1.55	32.91 ± 1.12
60	1/60	25	5	87.44 ± 1.25	56.65 ± 2.73	41.67 ± 0.85
50	1/40	100	4	47.40 ± 0.35	15.19 ± 0.78	23.69 ± 0.28
40	1/60	75	5	92.33 ± 0.32	48.27 ± .50	46.59 ± 0.14
Control by S-L extraction (Yield = 14.9%)				49.35 ± 2.06 ^a^	35.71 ± 0.19 ^a^	26.41 ± 1.88 ^a^
Optimum by S-L Extract. (Yield = 16.8%)				122.34 ± 2.35 ^b^	88.86 ± 0.78 ^b^	62.57 ± 3.37 ^b^

where TP is total polyphenol TF is total flavonoid and TF3 is total flavan-3-ol. S-L is the solid–liquid extraction. Means (*n* = 3) within a column (comparison of same bioactive compound family) with different letters were significantly different by ANOVA One-way Tukey (*p* < 0.05). For more information see the methodology section.

**Table 3:** Kinetic models used for the fitting for total polyphenol, total flavonoids, and total flavan-3-ols content from cocoa beans.

Model		Parameters for TP	Parameters for TF	Parameters for TF3	Ref.
**nth order (1)**	$\;C\left(t\right)=kt^{n}\;$	k=55.64 n=0.16	k=46.63 n=0.13	k=37.03 n=0.11	[26]
	*r* RMSE	0.93 8.23	0.90 5.46	0.90 4.19	
**Page (2)**	$\;C\left(t\right)=e^{kt^{n}}\;$	k=4.06 n=0.03	k=3.87 n=0.03	k=3.64 n=0.02	[23]
	*r* RMSE	0.93 8.52	0.89 5.55	0.89 4.22	
**Peleg (3)**	$\;C\left(t\right)=\frac{t}{k_{1}+k_{2}t}\;$	$\;k_{1}=0.03\;$ $k_{2}=8.1\times 10^{-3}$	$\;k_{1}=0.03\;$ $k_{2}=0.01$	$\;k_{1}=0.03\;$ $k_{2}=0.01$	[24]
	*r* RMSE	0.98 0.71	0.99 0.62	0.99 0.67	
**Weibull-type (4)**	$\;C\left(t\right)=C_{0}e^{kt^{n}}\;$	$\;C_{0}=1.7\times 10^{-8}\;$ k=21.92 $n=6.9\times 10^{-3}$	$\;C_{0}=2.8\times 10^{-7}\;$ k=13.17 $n=6.4\times 10^{-3}$	$\;C_{0}=2.0\times 10^{-7}\;$ k=19.03 $n=5.8\times 10^{-3}$	[22]
	*r* RMSE	0.93 8.33	0.89 5.51	0.90 4.32	
**Mincher and Minkov (5)**	$\;C\left(t\right)=A-Be^{-kt}\;$	A=96.94 B=96.94 k=289.60	A=72.29 B=72.29 k=103.69	A=55.07 B=55.07 k=117.03	[26]
	*r* RMSE	0.64 22.06	0.68 12.47	0.71 8.35	
**Pseudo first order (6)**	$\;C\left(t\right)=C_{\infty }-\frac{C_{\infty }}{e^{kt+a}}\;$	$\;C_{\infty }=122.34\;$ k=0.11 a=0.124	$\;C_{\infty }=88.87\;$ k=0.18 a=0.024	$\;C_{\infty }=62.57\;$ k=0.25 a=0.020	[25]
	*r* RMSE	0.98 6.49	0.98 5.59	0.99 2.29	

where TP is total polyphenol (mgGAE/g) TF is total flavonoid (mgECE/g), and TF3 is total flavan-3-ol (mgECE/g).

**Table 4:** Methylxanthine and procyanidin concentration, and characterization of cocoa beans using a HPLC-DAD-ESI-MS/MS method.

Compound	Reverse Phase		Ionization	Predicted Mass (mau)	Observed Mass (mau)	Error (mau)	HPLC-ESI-MS^n^
	Retention Time (min)	Concentration * (ppm)					MS^2^ Fragment (*m/z*)
Theobromine	1.42	7.78 ± 0.01	[M + H]^+^	181.07	181.8	0.73	137.5, 110.5
Caffeine	5.01	2.65 ± 0.02	[M + H]^+^	195.08	195.5	0.42	158.4, 138.7
Catechin	2.16	0.62 ± 0.01	[M − H]^+^	291.08	292.3	1.22	273.3, 165.3, 139.4, 123.7
Epicatechin	5.46	7.30 ± 0.10	[M − H]^+^	291.08	292.3	1.22	273.3, 165.3, 139.3, 123.6
Dimer B2	6.22	4.06 ± 0.03	[M − H]^−^	577.14	577.4	0.26	451.2, 425.1, 289.1, 271.1
Trimer C1	12.91	11.99 ± 0.25	[M − H]^−^	865.19	865.4	0.21	695.2, 577.2, 451.0, 289.0
Tetramer D	16.81	9.33 ± 0.40	[M − H]^−^	1153.26	1153.6	0.34	1027.3, 865.3, 739.2, 577.1
Pentamer	19.91	6.81 ± 0.52	[M − H]^−^	1441.33	1441.3	0.03	1153.3, 865.2, 691.6, 574.3
Hexamer	20.85	1.81 ± 0.01	[M − H]^−^	1729.38	1729.3	0.08	1534.0, 1153.3, 865.2, 574.2
Heptamer	22.51	ND	[M − H]^−^	2017.45	2019.3	1.85	1153.4, 995.3,851.3, 574.3

Data expressed as means of triplicate experiments. * Concentration expressed as mg polyphenol per g cocoa beans (dry weight basis).

## Supplementary Materials

The following are available online at https://www.mdpi.com/2076-3921/9/5/364/s1, Table S1: ANOVA for TP, TF, and TF3 through 24 surface design + central points+ start points.

## References

[1] Pedan V., Fischer N., Rohn S. (2016). An online NP-HPLC-DPPH method for the determination of the antioxidant activity of condensed polyphenols in cocoa. Food Res. Int..

[2] Batista N.N., de Andrade D.P., Ramos C.L., Dias D.R., Schwan R.F. (2016). Antioxidant capacity of cocoa beans and chocolate assessed by FTIR. Food Res. Int..

[3] Toro-Uribe S., Montero L., López-Giraldo L., Ibáñez E., Herrero M. (2018). Characterization of secondary metabolites from green cocoa beans using focusing-modulated comprehensive two-dimensional liquid chromatography coupled to tandem mass spectrometry. Anal. Chim. Acta.

[4] Pérez-Jiménez J., Neveu V., Vos F., Scalbert A. (2010). Identification of the 100 richest dietary sources of polyphenols: An application of the Phenol-Explorer database. Eur. J. Clin. Nutr..

[5] Presidencia de la República de Colombia Decreto Ley 896 de 2017 “Programa Nacional Integral de Sustitución de Cultivos de uso ilícito PNIS”. http://www.indepaz.org.co/wp-content/uploads/2017/05/Decreto-896-del-29-de-Mayo-de-2017-1.pdf.

[6] Quiroz-Reyes C.N., Aguilar-Mendez M.A., Ramírez-Ortíz M.E., Ronquillo-De Jesus E. (2013). Comparative study of ultrasound and maceration techniques for the extraction of polyphenols from cocoa beans (*Theobroma cacao* L.). Rev. Mex. Ing. Química.

[7] Routray W., Orsat V. (2012). Microwave-Assisted Extraction of Flavonoids: A Review. Food Bioprocess Technol..

[8] Okiyama D.C.G., Soares I.D., Cuevas M.S., Crevelin E.J., Moraes L.A.B., Melo M.P., Oliveira A.L., Rodrigues C.E.C. (2018). Pressurized liquid extraction of flavanols and alkaloids from cocoa bean shell using ethanol as solvent. Food Res. Int..

[9] Servent A., Boulanger R., Davrieux F., Pinot M.N., Tardan E., Forestier-Chiron N., Hue C. (2018). Assessment of cocoa (*Theobroma cacao* L.) butter content and composition throughout fermentations. Food Res. Int..

[10] Rodríguez-Carrasco Y., Gaspari A., Graziani G., Sandini A., Ritieni A. (2018). Fast analysis of polyphenols and alkaloids in cocoa-based products by ultra-high performance liquid chromatography and Orbitrap high resolution mass spectrometry (UHPLC-Q-Orbitrap-MS/MS). Food Res. Int..

[11] Żyżelewicz D., Budryn G., Oracz J., Antolak H., Kręgiel D., Kaczmarska M. (2018). The effect on bioactive components and characteristics of chocolate by functionalization with raw cocoa beans. Food Res. Int..

[12] Schinella G., Mosca S., Cienfuegos-Jovellanos E., Pasamar M.Á., Muguerza B., Ramón D., Ríos J.L. (2010). Antioxidant properties of polyphenol-rich cocoa products industrially processed. Food Res. Int..

[13] Pons-Andreu J.-V., Cienfuegos-Jovellanos E., Ibarra A. (2008). Process for Producing Cocoa Polyphenol Concentrate. U.S. Patent.

[14] Pinelo M., Sineiro J., Núñez M.J. (2006). Mass transfer during continuous solid-liquid extraction of antioxidants from grape byproducts. J. Food Eng..

[15] Carrillo L.C., Londoño-Londoño J., Gil A. (2014). Comparison of polyphenol, methylxanthines and antioxidant activity in Theobroma cacao beans from different cocoa-growing areas in Colombia. Food Res. Int..

[16] Ioannone F., Di Mattia C.D., De Gregorio M., Sergi M., Serafini M., Sacchetti G. (2015). Flavanols, proanthocyanidins and antioxidant activity changes during cocoa (*Theobroma cacao* L.) roasting as affected by temperature and time of processing. Food Chem..

[17] Patras M.A., Milev B.P., Vrancken G., Kuhnert N. (2014). Identification of novel cocoa flavonoids from raw fermented cocoa beans by HPLC-MSn. Food Res. Int..

[18] Chew K.K., Khoo M.Z., Ng S.Y., Thoo Y.Y., Aida W.M.W., Ho C.W. (2011). Effect of ethanol concentration, extraction time and extraction temperature on the recovery of phenolic compounds and antioxidant capacity of Orthosiphon stamineus extracts. Int. Food Res. J..

[19] Singleton V.L., Orthofer R., Lamuela-Raventós R.M. (1998). Analysis of total phenols and other oxidation substrates and antioxidants by means of folin-ciocalteu reagent. Methods Enzymol..

[20] Zhishen J., Mengcheng T., Jianming W. (1999). The determination of flavonoid contents in mulberry and their scavenging effects on superoxide radicals. Food Chem..

[21] Sun B., Ricardo-da-Silva J.M., Spranger I. (1998). Critical Factors of Vanillin Assay for Catechins and Proanthocyanidins. J. Agric. Food Chem..

[22] Amendola D., De Faveri D.M., Spigno G. (2010). Grape marc phenolics: Extraction kinetics, quality and stability of extracts. J. Food Eng..

[23] Doymaz İ., İsmail O. (2011). Drying characteristics of sweet cherry. Food Bioprod. Process..

[24] Peleg M. (1988). An Empirical Model for the Description of Moisture Sorption Curves. J. Food Sci..

[25] Spiro M., Jago D.S. (1982). Kinetics and equilibria of tea infu- sion. Part 3. Rotating disc experiments interpreted by a steady state model. J. Chem. Soc. Faraday Trans. 1.

[26] Sant’Anna V., Brandelli A., Marczak L.D.F., Tessaro I.C. (2012). Kinetic modeling of total polyphenol extraction from grape marc and characterization of the extracts. Sep. Purif. Technol..

[27] Toro-Uribe S., López-Giraldo L.J., Decker E.A. (2018). Relationship between the physiochemical properties of cocoa procyanidins and their ability to inhibit lipid oxidation in liposomes. J. Agric. Food Chem..

[28] Huang D., Ou B., Hampsch-Woodill M., Flanagan J.A., Prior R.L. (2002). High-throughput assay of oxygen radical absorbance capacity (ORAC) using a multichannel liquid handling system coupled with a microplate fluorescence reader in 96-well format. J. Agric. Food Chem..

[29] Brand-Williams W., Cuvelier M.E., Berset C. (1995). Use of a free radical method to evaluate antioxidant activity. LWT Food Sci. Technol..

[30] Kothe L., Zimmermann B.F., Galensa R. (2013). Temperature influences epimerization and composition of flavanol monomers, dimers and trimers during cocoa bean roasting. Food Chem..

[31] Sun Y., Liu D., Chen J., Ye X., Yu D. (2011). Effects of different factors of ultrasound treatment on the extraction yield of the all-trans-B-carotene from citrus peels. Ultrason. Sonochem..

[32] Goula A.M. (2013). Ultrasound-assisted extraction of pomegranate seed oil—Kinetic modeling. J. Food Eng..

[33] Hoskin J.M., Dimick P.S., Daniels R.R. (1980). Scanning Electron Microscopy of the Theobroma cacao Seed. J. Food Sci..

[34] Lopez A.S., Dimick P.S., Walsh R.M. (1987). Scanning Electron Microscopy Studies of the Cellular Changes in Raw, Fermented and Dried Cocoa. Food Struct..

[35] Rostagno M.A., Palma M., Barroso C.G. (2003). Ultrasound-assisted extraction of soy isoflavones. J. Chromatogr. A.

[36] Cacace J.E., Mazza G. (2003). Mass transfer process during extraction of phenolic compounds from milled berries. J. Food Eng..

[37] Mokrani A., Madani K. (2016). Effect of solvent, time and temperature on the extraction of phenolic compounds and antioxidant capacity of peach (*Prunus persica* L.) fruit. Sep. Purif. Technol..

[38] Rakotondramasy-Rabesiaka L., Havet J. (2010). Estimation of effective diffusion and transfer rate during the protopine extraction process from *Fumaria officinalis* L. Sep. Purif. Technol..

[39] Datta C., Dutta A., Dutta D., Chaudhuri S. (2011). Adsorption of polyphenols from ginger rhizomes on an anion exchange resin Amberlite IR-400—Study on effect of pH and temperature. Procedia Food Sci..

[40] Galvan D’Alessandro L., Kriaa K., Nikov I., Dimitrov K. (2012). Ultrasound assisted extraction of polyphenols from black chokeberry. Sep. Purif. Technol..

[41] Othman S.N.S., Mustapa A.N., Ku Hamid K.H. (2020). Extraction of polyphenols from Clinacanthus nutans Lindau (*C. nutans*) by vacuum solvent-free microwave extraction (V-SFME). Chem. Eng. Commun..

[42] Bucić-Kojić A., Planinić M., Tomas S., Bilić M., Velić D. (2007). Study of solid-liquid extraction kinectics of total polyphenols from grape seeds. J. Food Eng..

[43] Tomas-Barberán F.A., Cienfuegos-Jovellanos E., Marín A., Muguerza B., Gil-Izquierdo A., Cerdá B., Zafrilla P., Morillas J., Mulero J., Ibarra A. (2007). A new process to develop a cocoa powder with higher flavonoid monomer content and enhanced bioavailability in healthy humans. J. Agric. Food Chem..

[44] Maisuthisakul P. (2009). Antioxidant Potential and Phenolic Constituents of Mango Seed. Kasetsart J. Nat. Sci..

[45] Crozier S.J., Hurst W.J., Watson R.R., Preedy V.R., Zibadi S. (2013). Cocoa Polyphenols and Cardiovascular Health. Polyphenols in Human Health and Disease.

[46] Racine K.C., Wiersema B.D., Griffin L.E., Essenmacher L.A., Lee A.H., Hopfer H., Lambert J.D., Stewart A.C., Neilson A.P. (2019). Flavanol polymerization is a superior predictor of α-glucosidase inhibitory activity compared to flavanol or total polyphenol concentrations in cocoas prepared by variations in controlled fermentation and roasting of the same raw cocoa beans. Antioxidants.

[47] Hasing M.H. (2004). Estudio de la Variación en los Contenidos de Polifenoles y Alcaloides, en Almendras de Cacao por Efecto de los Procesos de Fermentación y Tostado. Ph.D. Thesis.

[48] Fernández-Romero E., Chavez-Quintana S.G., Siche R., Castro-Alayo E.M., Cardenas-Toro F.P. (2020). The kinetics of total phenolic content and monomeric Flavan-3-ols during the roasting process of Criollo Cocoa. Antioxidants.

[49] Niemenak N., Rohsius C., Elwers S., Ndoumou D., Liebereri R. (2006). Comparative study of different cocoa (*Theobroma cacao* L.) clones in terms of their phenolics and anthocyanins contents. J. Food Compos. Anal..

[50] Rockenbach I.I., Jungfer E., Ritter C., Santiago-Schübel B., Thiele B., Fett R., Galensa R. (2012). Characterization of flavan-3-ols in seeds of grape pomace by CE, HPLC-DAD-MS n and LC-ESI-FTICR-MS. Food Res. Int..

[51] Hurst W.J., Payne M.J., Miller K.B., Stuart D.A. (2009). Stability of cocoa antioxidants and flavan-3-ols over time. J. Agric. Food Chem..

[52] Summa C., Raposo F.C., McCourt J., Scalzo R.L., Wagner K.H., Elmadfa I., Anklam E. (2006). Effect of roasting on the radical scavenging activity of cocoa beans. Eur. Food Res. Technol..

