# Supplementary material for: Food-Safe Process for High Recovery of Flavonoids from Cocoa Beans: Antioxidant and HPLC-DAD-ESI-MS/MS Analysis
Source: Antioxidants (Basel). 2020 Apr 27;9(5):364. doi: 10.3390/antiox9050364 (PMC7278696; doi:10.3390/antiox9050364)
Supplement: Supplementary file 1 [file antioxidants-09-00364-s001.pdf]

Electronic Supplementary Material to:

Food-safe process for high recovery of flavonoids from cocoa beans: antioxidant and HPLC- DAD-ESI-MS/MS analysis

Said Toro-Uribe [1], Elena Ibañez [2], Eric A. Decker [3], Arley René Villamizar-Jaimes [4], Luis Javier López-Giraldo [1,*]

Table S1. ANOVA for TP, TF, and TF3 through 24 surface design + central points+ start points.

	Total Polyphenols (mgGAE/g)					Total Flavonoids (mgECE/g)					Total Flavan-3-ols (mgECE/g)				
	**SS**	**df**	**MS**	**F**	**p**	**SS**	**df**	**MS**	**F**	**p**	**SS**	**df**	**MS**	**F**	**p**
**T**	121.89	1	121.892	25.054	0.00002	145.61	1	145.612	25.0688	0.00002	331.817	1	331.817	67.2240	0.00000
**T^2**	24.53	1	24.534	5.043	0.03251	724.60	1	724.600	124.748	0.00000	6.457	1	6.457	1.3082	0.26207
**SS**	279.34	1	279.342	57.416	0.00000	2281.04	1	2281.037	392.706	0.00000	4.410	1	4.410	0.8934	0.35235
**SS^2**	283.53	1	283.529	58.277	0.00000	113.79	1	113.786	19.5896	0.00012	56.859	1	56.859	11.5193	0.00201
**EW**	765.38	1	765.383	157.31	0.00000	106.59	1	106.594	18.3514	0.00018	209.481	1	209.481	42.4395	0.00000
**EW^2**	5940.53	1	5940.52	1221.0	0.00000	5591.41	1	5591.412	962.625	0.00000	1710.30	1	1710.30	346.495	0.00000
**pH**	382.70	1	382.701	78.661	0.00000	134.49	1	134.493	23.1546	0.00004	595.725	1	595.725	120.690	0.00000
**pH^2**	227.97	1	227.967	46.857	0.00000	5.92	1	5.920	1.0193	0.32104	5.672	1	5.672	1.1492	0.29256
**T×SS**	3.89	1	3.894	0.800	0.37832	43.38	1	43.376	7.4676	0.01058	98.727	1	98.727	20.0015	0.00011
**T×EW**	28.04	1	28.045	5.764	0.02299	9.23	1	9.226	1.5883	0.21761	282.789	1	282.789	57.2912	0.00000
**T×pH**	0.03	1	0.026	0.005	0.94216	1.21	1	1.210	0.2084	0.65143	6.530	1	6.530	1.3229	0.25946
**SS×EW**	4.75	1	4.751	0.977	0.33121	511.66	1	511.657	88.0875	0.00000	46.878	1	46.878	9.4972	0.00448
**SS×pH**	3.76	1	3.764	0.774	0.38632	1.59	1	1.587	0.2733	0.60510	26.738	1	26.738	5.4170	0.02712
**EW×pH**	169.72	1	169.718	34.884	0.00000	3.97	1	3.972	0.6839	0.41500	130.251	1	130.251	26.3881	
**Error**	475.99	39	12.205			585.86	39	15.022			436.821	39	11.201		
**Total SS**	10988.6	53				12768.3	53				4622.14	53			

where SS is the sum of square, df the degree of freedom, MS the mean square, p is the value of confidence interval, T (temperature), SS (solute/solvent ratio), and EA (ethanol/water ratio). TP is total polyphenol, TF is total flavonoid, and TF3 is total flavan-3ol

Supplementary Equations

Predicted mathematical for TP, TF, and TF3 on cocoa beans, as follows:

Equation S1. Predicted model for total polyphenols content

$$Y_{TPC} = 104.36 + 0.01T - 2063.89\,SS + 36443.83\,SS^2 + 2.62\,EW - 0.02EW^2 - 19.01pH + 2.72\,pH^2 - 0.09EW * pH$$

Equation S2. Predicted model for total flavonoids content

$$Y_{TF} = 99.20 - 3.71T + 0.04\,T^2 + 23087.14\,SS^2 + 1.33\,EW - 0.02EW^2 + 19.19\,SS * pH$$

Equation S3. Predicted model for a total flavan-3-ol content

$$Y_{TF3} = 16320.24\,SS^2 - 0.01\,EW^2 - 21.08\,T * SS + 0.01\,T * EW + 5.81\,SS * pH + 0.08\,EW * pH$$
